# Awareness of HPV-related cancers and the HPV vaccination programme in Scotland: a cross-sectional study of gender, deprivation and knowledge gaps

**DOI:** 10.1093/pubmed/fdaf136

**Published:** 2025-11-04

**Authors:** Huyen Nguyen-Thanh, Leah Marshall, Hannah Wilson, Kirsty Stewart, Kimberley Kavanagh, Annette Sorensen

**Affiliations:** Department of Pharmacy, Vinmec Central Park International Hospital, Binh Thanh District, Ho Chi Minh, Vietnam; Department of Mathematics and Statistics, University of Strathclyde, Glasgow G1 1XH, UK; Strathclyde Institute of Pharmacy and Biomedical Sciences, University of Strathclyde, Glasgow G4 0RE, UK; Strathclyde Institute of Pharmacy and Biomedical Sciences, University of Strathclyde, Glasgow G4 0RE, UK; Public Health Scotland, Glasgow, G2 7ER, United Kingdom; Department of Mathematics and Statistics, University of Strathclyde, Glasgow G1 1XH, UK; Strathclyde Institute of Pharmacy and Biomedical Sciences, University of Strathclyde, Glasgow G4 0RE, UK

**Keywords:** HPV vaccination, health inequalities, cancer prevention, public health, socioeconomic

## Abstract

**Background:**

Scotland’s human papillomavirus (HPV) vaccination programme includes boys, yet awareness of male-associated cancers and eligibility remains unclear. Awareness may differ by gender and socioeconomic status, contributing to inequalities and declining uptake. This study assessed knowledge of HPV, HPV-related cancers and the vaccination programme.

**Methods:**

A cross-sectional online survey (*n* = 1052) was conducted in 2023. Descriptive statistics and logistic regression explored gender- and deprivation-related differences in awareness of HPV, HPV-related cancers and the vaccination programme.

**Results:**

Overall, 80.6% had heard of HPV, with higher awareness among females (89.2%) than males (67.3%, *P* < .001) and in less deprived areas (88.8% vs. 79.8%, *P* = .004). Cervical cancer was recognized (78.9% females, 54.8% males), while awareness of non-cervical cancers was low, especially oropharyngeal (<10%). Fewer than half (48.9%) knew boys are included in Scotland’s programme. Vaccine awareness strongly predicted knowledge of male cancers (adjusted odds ratio [aOR] = 4.41) and the boys’ programme (aOR = 10.67). Despite knowledge gaps, willingness to vaccinate children remained high (>92%).

**Conclusions:**

Awareness of male HPV-related cancers and the boys’ vaccination programme remains limited, with gender and socioeconomic disparities. A ‘knowledge-action gap’ was evident, with support despite poor understanding. Targeted, inclusive education is needed to reduce inequalities and sustain confidence.

## Introduction

Human papillomavirus (HPV) is a widespread sexually transmitted infection linked not only to cervical cancer^1^ but also to penile, anal, and oropharyngeal cancers affecting both women and men.^2^ However, public awareness of these non-cervical HPV-related cancers remains low, particularly among men, potentially limiting preventive behaviours and vaccine uptake.^3^

In Scotland, the HPV vaccine is routinely offered through school-based programmes to all S1 pupils (11–13 years old). Pupils who miss vaccination in S1 can receive it in subsequent school years. Since 2019, the programme, introduced for girls in 2008, has included boys^4^ reflecting recognition of HPVs health impact and the value of gender-neutral vaccination strategies. Awareness and knowledge of HPV are critical to vaccine acceptance and uptake,^5^ but studies focusing on non-cervical cancers remain limited. A recent systematic review found only 8 of 86 studies from high-income countries examined awareness of non-cervical HPV-related cancers.^3^ Reported awareness was generally low (17%–30%) with females consistently more informed than males. Although countries such as Australia and Finland reported higher overall awareness, differences in vaccine delivery models (school- vs. clinic-based) failed to explain variation, highlighting the importance of national education and communication strategies.

No studies have examined awareness of HPV-related cancers in Scotland. This is concerning given declining vaccine uptake. In 2023–24, only 68.7% of S1 boys received their first dose, down from 78.7% in 2019–20.^6^^,^^7^ Among girls, uptake was 74.4%, revealing a gender disparity of 5.7%.^6^ Vaccine uptake among girls has also declined and remains below pre-COVID-19 levels.^6^ Socioeconomic disparities have widened: among S4 pupils, the difference between most and least deprived groups increased from 3.1% in 2019–20 to 12.7% (79.9% vs. 92.6%) in 2023–24.^6^ Although the impact of deprivation on uptake is well-established, its influence on HPV awareness is poorly understood.^8^

As HPV vaccination is voluntary worldwide, public awareness and attitudes are key to uptake.^9^ Factors such as parental education, prior vaccine experience, and trust in efficacy influence acceptance,^10^ but these may differ for boys given the historically female-centric focus of HPV prevention. This study is the first to assess awareness of HPV-related cancers and the HPV vaccination programme for girls and boys in Scotland and the first to analyse variation by socioeconomic status using the Scottish Index of Multiple Deprivation (SIMD). It fills a national gap and contributes to international evidence on awareness disparities despite universal school-based delivery.

## Methods

### Study design and participants

This cross-sectional, population-based survey assessed awareness of HPV, its cancer links and the HPV vaccination programme in Scotland. Survey questions were developed based on a review of existing literature.^5^^,^^11–13^ A pilot survey with 100 participants from the target population was conducted to assess reliability, with consistent response patterns indicating questions were generally clear. For content validity, the questionnaire was reviewed for relevance and clarity by cancer researchers with expertise in HPV. Revisions were made based on feedback before full deployment. The final survey was distributed via social media and the University of Strathclyde’s teaching platform between October and December 2023, yielding 1052 responses. Convenience sampling without a defined sampling frame prevented calculation of a response rate. All data were self-reported and anonymous. Participation was voluntary, with assurances of confidentiality and all data were securely stored. The study received ethical approval from the SIPBS Ethics Committee.

### Outcome variables and measures

The primary outcome was general HPV awareness, measured by a binary yes/no response to having heard of HPV. Secondary outcomes included awareness of specific HPV-related cancers and the HPV vaccine. Awareness of female cancers was assessed for cervical, oropharyngeal, vulvar, vaginal and anal cancers, while male cancer awareness covered oropharyngeal, penile and anal cancers. Participants were first asked to name HPV-related cancers, then selected from a list that included the same cancers assessed for awareness, alongside cancers not currently established as HPV-related. Additional measures included knowledge of specific HPV-cancer associations, such as whether participants knew over 99% of cervical cancer cases and 70% of throat cancer (oropharyngeal cancer) are estimated to be attributed to HPV in the UK.

Two primary dichotomous variables (‘Yes’ or ‘No’) were derived from the survey responses and used in univariable and multivariable logistic regression analysis to assess factors associated with awareness of HPV-related cancers in males and the HPV vaccination programme for boys:

Male HPV-related cancers awareness: the participant correctly identifying at least one HPV-related male cancer from a multiple-choice list or free-text response.

HPV vaccination programme awareness: the participant knew that the HPV vaccine is offered to all boys in secondary schools in Scotland.

Additional outcomes included general vaccine awareness and personal vaccination status. Awareness of the national vaccination programme was assessed for both girls and boys, alongside self-rated HPV vaccine knowledge (scored 1–10). Participants also reported willingness to consent to HPV vaccination for their children and reasons influencing their decision. To assess socioeconomic influences on awareness, respondents provided their postcode, which was mapped to SIMD. SIMD ranks small areas in Scotland into five quantiles based on income, employment, education, health, access to services, crime and housing.^14^ Quintile 1 represents the 20% most deprived and Quintile 5 represents the 20% least deprived areas. Other socioeconomic indicators included employment status, education level and current student status. Demographic data were collected on age, gender, sexual orientation, ethnicity and parental status.

### Statistical analysis

Descriptive statistics and cross-tabulation were used to calculate frequencies and percentages of participant characteristics. A linear logistic regression model tested trends in HPV-related awareness across SIMD quintiles. Gender differences were assessed using Pearson Chi-squared or Fisher’s exact tests. Univariable and multivariable logistic regression analyses explored factors associated with male HPV-related cancers and awareness of the boys vaccination programme. Odds ratios (OR) and adjusted odds ratios (aOR), which estimate associations between factors and outcomes while controlling for other variables, were reported with 95% confidence intervals (CI). Analyses were conducted in R Statistical Software version 4.3.1.^15^ Results were considered statistically significant at the 5% level (*P* < .05).

## Results

### Sociodemographic characteristics

Table 1 summarizes the characteristics of 1052 participants. The sample was predominant female (78.3%), with most aged 17–24 (39.2%) or over 45 (27.0%). Among 17 to 24-year-olds, 90.3% of females reported receiving the HPV vaccine compared to 38.8% of males. Most identified as Scottish, English, Welsh, Northern Irish, or British (91.0%) and heterosexual (84.9%). Of those in education (41.3%), 85.4% were pursuing university degrees and 51.8% were in health-related programmes. Among employed participants (80.5%), 14.2% worked in health-related fields. SIMD showed 22.1% resided in the least deprived and 8.9% in the most deprived areas.

**Table 1:** Socio-demographic characteristics of respondents.

**Characteristics**	**Female (824)** **(*N*, %)**	**Male (228)** **(*N*, %)**	**Total (1052)** **(*N*, %)**
**Vaccination status**			
Vaccinated	341 (48.1)	44 (21.5)	385 (42.1)
Unvaccinated	368 (51.9)	161 (78.5)	529 (57.9)
**Age**			
Under 17	15 (1.8)	2 (0.9)	17 (1.6)
17–24	321 (39.0)	91 (40.1)	412 (39.2)
25–34	109 (13.2)	37 (16.3)	146 (13.9)
35–45	155 (18.8)	37 (16.3)	192 (18.3)
Above 45	224 (27.2)	60 (26.4)	284 (27.0)
**Sexual orientation**			
Heterosexual	698 (85.2)	190 (83.7)	888 (84.9)
Bisexual	65 (7.9)	11 (4.8)	76 (7.3)
Homosexual	18 (2.2)	24 (10.6)	42 (4.0)
Other	18 (2.2)	0 (0.0)	18 (1.7)
Prefer not to say	20 (2.4)	2 (0.9)	22 (2.1)
**Ethnicity**			
Scottish, English, Welsh, Northern Irish or British	753 (91.6)	203 (89.0)	956 (91.0)
Other White background	18 (2.2)	8 (3.5)	26 (2.5)
Mixed or Multiple ethnic background	5 (0.6)	1 (0.4)	6 (0.6)
Asian or Asian British	27 (3.3)	10 (4.4)	37 (3.5)
Black, African, Caribbean, or Black British	10 (1.2)	2 (0.9)	12 (1.1)
Others ethnic group	6 (0.7)	4 (1.8)	10 (1.0)
Prefer not to say	3 (0.4)	0 (0.0)	3 (0.3)
**Currently in education**			
Yes	344 (42.0)	88 (38.8)	432 (41.3)
No	476 (58.0)	139 (61.2)	615 (58.7)
**Level of education (Currently in education = Yes)**			
School	24 (7.3)	3 (3.4)	27 (6.5)
College	8 (2.4)	2 (2.3)	10 (2.4)
University	278 (84.2)	78 (89.6)	356 (85.4)
Postgraduate study	20 (6.1)	4 (4.7)	24 (5.7)
**Health-related degree (currently in education = Yes)**			
Yes	173 (50.9)	48 (55.2)	221 (51.8)
No	167 (49.1)	39 (44.8)	206 (48.2)
**Employment status**			
Full time	328 (40.7)	130 (57.8)	458 (44.4)
Part time	325 (40.3)	47 (20.9)	372 (36.1)
Retired	104 (12.9)	37 (16.4)	141 (13.7)
Unemployed	49 (6.1)	11 (4.9)	60 (5.8)
**Health-related employment (employment status = full time/part time)**			
Yes	105 (16.1)	13 (7.3)	118 (14.2)
No	548 (83.9)	164 (92.7)	712 (85.8)
**Having children**			
Yes	330 (40.0)	79 (34.6)	409 (38.9)
No	401 (48.7)	128 (56.1)	529 (50.3)
**Scottish Index of Multiple Deprivation (SIMD)**			
Quintile 1 (most deprived)	70 (8.5)	24 (10.5)	94 (8.9)
Quintile 2	110 (13.3)	36 (15.8)	146 (13.9)
Quintile 3	104 (12.6)	33 (14.5)	137 (13.1)
Quintile 4	122 (14.8)	37 (16.2)	159 (15.1)
Quintile 5 (least deprived)	192 (23.3)	41 (18.0)	233 (22.1)

### Awareness and knowledge of HPV and HPV-related cancers

Table 2 shows awareness by SIMD and gender. Overall, 80.6% had heard of HPV with higher awareness in less deprived areas (88.8% quintile 5 vs. 79.8% quintile 1, *P* = .004) and among females (89.2% vs. 67.3% males, *P* < .001). Cervical cancer was most widely recognized (78.9% females, 54.8% males, *P* < .001), but only 32.8% of females and 18.0% of males identified HPV as the main risk factor (*P* < .001). Awareness of vulvar and vaginal cancer was moderate (40.0% females, 29.4% males, *P* = .004). Anal cancer awareness was low across genders (11.8% females, 10.5% males, *P* = .686), as was oropharyngeal cancer awareness (4.1% females, 3.5% males, *P* = .818). SIMD showed no significant influence.

**Table 2:** Awareness and knowledge of HPV-related cancers and vaccine.

	**Scottish Index of Multiple Deprivation (SIMD)**						**Gender**		
**Variables**	**Quintile 1** **(*N*, %)**	**Quintile 2** **(*N*, %)**	**Quintile 3** **(*N*, %)**	**Quintile 4** **(*N*, %)**	**Quintile 5** **(*N*, %)**	*P*-value*	**Female** **(*N*, %)**	**Male** **(*N*, %)**	*P*-value^*^
**Heard of HPV**									
Yes	75 (79.8)	111 (76.0)	115 (83.9)	137 (86.2)	207 (88.8)	.004	700 (89.2)	148 (67.3)	<.001
No	19 (20.2)	35 (24.0)	22 (16.1)	22 (13.8)	26 (11.2)		85 (10.8)	72 (32.7)	
**Female HPV-related cancers awareness**									
*Cervical cancer*									
Yes	75 (79.8)	106 (72.6)	111 (81.0)	118 (74.2)	191 (82.0)	.142	650 (78.9)	125 (54.8)	<.001
No	19 (20.2)	40 (27.4)	26 (19.0)	41 (25.8)	42 (18.0)		174 (21.1)	103 (45.2)	
*Oropharyngeal cancer (Throat cancer)*									
Yes	2 (2.1)	7 (4.8)	4 (2.9)	5 (3.1)	17 (7.3)	.190	34 (4.1)	8 (3.5)	.818
No	92 (97.9)	139 (95.2)	133 (97.1)	154 (96.9)	216 (92.7)		790 (95.9)	220 (96.5)	
*Vulvar and Vaginal cancer*									
Yes	39 (41.5)	54 (37.0)	62 (45.3)	64 (40.3)	97 (41.6)	.785	330 (40.0)	67 (29.4)	.004
No	55 (58.5)	92 (63.0)	75 (54.7)	95 (59.7)	136 (58.4)		494 (60.0)	161 (70.6)	
*Anal cancer*									
Yes	12 (12.8)	21 (14.4)	16 (11.7)	18 (11.3)	30 (12.9)	.752	97 (11.8)	24 (10.5)	.686
No	82 (87.2)	125 (85.6)	121 (88.3)	141 (88.7)	203 (87.1)		727 (88.2)	204 (89.5)	
**Male HPV-related cancers awareness**									
*Oropharyngeal cancer (Throat cancer)*									
Yes	6 (6.4)	11 (7.5)	12 (8.8)	14 (8.8)	25 (10.7)	.727	70 (8.5)	15 (6.6)	.422
No	88 (93.6)	135 (92.5)	125 (91.2)	145 (91.2)	208 (89.3)		754 (91.5)	213 (93.4)	
*Penile cancer*									
Yes	30 (31.9)	45 (30.8)	37 (27.0)	52 (32.7)	86 (36.9)	.356	252 (30.6)	52 (22.8)	.027
No	64 (68.1)	101 (69.2)	100 (73.0)	107 (67.3)	147 (63.1)		572 (69.4)	176 (77.2)	
*Anal cancer*									
Yes	18 (19.1)	30 (20.5)	21 (15.3)	36 (22.6)	44 (18.9)	.896	147 (17.8)	36 (15.8)	.533
No	76 (80.9)	116 (79.5)	116 (84.7)	123 (77.4)	189 (81.1)		677 (82.2)	192 (84.2)	
**Knew that over 99% of cervical cancer cases in the UK are caused by HPV infection**									
Yes	21 (23.1)	30 (22.2)	44 (32.8)	38 (25.5)	72 (32.3)	.083	243 (32.8)	37 (18.0)	<.001
No	70 (76.9)	105 (77.8)	90 (67.2)	111 (74.5)	151 (67.7)		498 (67.2)	169 (82.0)	
**Knew that an estimated 70% of throat cancers are attributed to HPV infection**									
Yes	9 (9.9)	7 (5.3)	15 (11.2)	12 (7.9)	25 (11.4)	.435	74 (10.1)	15 (7.2)	.258
No	82 (90.1)	125 (94.7)	119 (88.8)	140 (92.1)	194 (88.6)		657 (89.9)	193 (92.8)	
**Heard of the HPV vaccine**									
Yes	68 (73.1)	105 (72.9)	120 (87.6)	130 (83.3)	199 (86.1)	<.001	682 (87.7)	133 (61.0)	<.001
No	25 (26.9)	39 (27.1)	17 (12.4)	26 (16.7)	32 (13.9)		96 (12.3)	85 (39.0)	
**Received the HPV vaccination for themself**									
Yes	38 (42.7)	56 (44.1)	61 (46.9)	67 (45.0)	81 (37.9)	.517	341 (48.1)	44 (21.5)	<.001
No	51 (57.3)	71 (55.9)	69 (53.1)	82 (55.0)	133 (62.1)		368 (51.9)	161 (78.5)	
**Knew that as part of a national vaccination programme, the HPV vaccine is offered to all girls in secondary schools in Scotland**									
Yes	71 (75.5)	107 (73.8)	112 (81.8)	128 (81.0)	192 (82.8)	.047	682 (87.1)	122 (55.7)	<.001
No	23 (24.5)	38 (26.2)	25 (18.2)	30 (19.0)	40 (17.2)		101 (12.9)	97 (44.3)	
**Knew that as part of a national vaccination programme, the HPV vaccine is offered to all boys in secondary schools in Scotland**									
Yes	28 (31.1)	44 (32.6)	57 (41.9)	52 (34.2)	96 (42.5)	.071	303 (40.6)	52 (25.0)	<.001
No	62 (68.9)	91 (67.4)	79 (58.1)	100 (65.8)	130 (57.5)		443 (59.4)	156 (75.0)	
**Self-rated HPV vaccine knowledge**									
1–4	33 (40.2)	57 (50.0)	40 (32.8)	61 (43.0)	74 (36.3)	.028	243 (36.0)	92 (52.6)	<.001
5–7	44 (53.7)	48 (42.1)	67 (54.9)	57 (40.1)	104 (51.0)		343 (50.8)	67 (38.3)	
8–10	5 (6.1)	9 (7.9)	15 (12.3)	24 (16.9)	26 (12.7)		89 (13.2)	16 (9.1)	
**Consent the HPV vaccination for their children**									
Yes	82 (92.1)	123 (96.9)	129 (98.5)	148 (98.0)	207 (95.8)	.160	695 (97.1)	194 (94.6)	.144
No	7 (7.9)	4 (3.1)	2 (1.5)	3 (2.0)	9 (4.2)		21 (2.9)	11 (5.4)	

^*^Linear test of trend *P*-value.

For male-specific cancers, awareness was higher in females for penile cancer (30.6% vs. 22.8% males, *P* = .027). Awareness of anal cancer (17.8% females, 15.8% males, *P* = .533) and HPV’s role in oropharyngeal cancer remained lower (8.5% females, 6.6% males, *P* = .422). SIMD had no significant effect on awareness of male-specific HPV-related cancers. Only 10.1% of females and 7.2% of males knew HPV is implicated in around 70% of throat cancers (*P* = .258), with no significant SIMD variation.

Table 3 presents regressions analysis of factors associated with awareness of male HPV-related cancers. Awareness was higher among participants under 17 (OR = 2.51, 95% CI: 0.84–7.48, *P* = .007) and females (OR = 1.73, 95% CI: 1.24–2.42, *P* < .001). It was also higher among those in education (OR = 1.35, 95% CI: 1.04–1.76, *P* = .033), those who had heard of HPV (OR = 2.59, 95% CI: 1.73–3.97, *P* < .001) and those aware of HPV vaccine (OR = 3.06, 95% CI: 2.07–4.61, *P* < .001). A health-related degree or employment doubled the odds of being aware of at least one male HPV-related cancer (OR = 2.22, 95% CI: 1.43–3.49, *P* < .001). Multivariable models showed HPV vaccine awareness (aOR = 4.41, 95% CI: 2.59–7.50, *P* < .001), vaccine receipt (aOR = 3.87, 95% CI: 1.64–9.13, *P* = .002) and health-related background (aOR = 2.26, 95% CI: 1.41–3.62, *P* < .001) remained strong predictors. SIMD was significant (*P* = .011) but without a clear linear trend.

**Table 3:** Logistic regression analysis of male HPV-related cancers awareness.

		**Univariable analysis**		**Multivariable analysis**	
**Independent variables**		**Male HPV-related cancers awareness** **(‘Yes’ vs ‘No’)**	***P*-value**	**Male HPV-related cancers awareness** **(‘Yes’ vs ‘No’)**	***P*-value**
		**OR (95% CI)**		**aOR (95% CI)**	
Age **(Ref = ‘17–24’)**	Under 17	2.51 (0.84–7.48)	.007		
		25–34	0.52 (0.33–0.80)		
		35–45	0.71 (0.49–1.02)		
		Above 45	0.87 (0.63–1.21)		
Gender **(Ref = ‘Male’)**	Female	1.73 (1.24–2.42)	<.001		
Sexual orientation **(Ref = ‘Heterosexual’)**	Bisexual	1.19 (0.72–1.96)	.538		
	Homosexual	1.71 (0.92–3.21)			
	Other	0.93 (0.31–2.54)			
	Prefer not to say	0.93 (0.31–2.54)			
Ethnicity **(Ref = ‘Scottish, English, Welsh, Northern Irish or British’)**	Other White background	1.55 (0.66–3.67)	.310		
	Mixed or Multiple ethnic background	4.66 (0.59–94.49)			
	Asian or Asian British	1.55 (0.74–3.24)			
	Black, African, Caribbean, or Black British	2.33 (0.66–9.18)			
	Others ethnic group	1.24 (0.31–4.73)			
	Prefer not to say	0 (0, Inf)			
Currently in education **(Ref = ‘No’)**	Yes	1.35 (1.04–1.76)	.033		
Employment status **(Ref = ‘Unemployed’)**	Full time	0.58 (0.38–0.87)	.001	0.70 (0.45–1.10)	.012
	Part time	0.88 (0.58–1.34)		0.83 (0.52–1.31)	
	Retired	0.93 (0.49–1.77)		1.37 (0.68–2.79)	
Heard of HPV **(Ref = ‘No’)**	Yes	2.59 (1.73–3.97)	<.001		
Heard of HPV vaccine **(Ref = ‘No’)**	Yes	3.06 (2.07–4.61)	<.001	4.41 (2.59–7.50)	<.001
Have children **(Ref = ‘No’)**	Yes	0.95 (0.73–1.23)	0.854		
Received HPV vaccine **(Ref = ‘No’)**	Yes	1.30 (0.99–1.70)	.144	3.87 (1.64–9.13)	.002
Post-COVID-19 pandemic attitudes towards HPV vaccination **(Ref = ‘No—I have remained against HPV vaccination pre and post the pandemic’)**	No—I remain to support HPV vaccination pre and post pandemic	1.71 (0.95–3.20)	.057		
	Yes—I did support HPV vaccination pre pandemic and now do not support HPV vaccination post pandemic	1.64 (0.58–4.62)			
	Yes—I was against HPV vaccination pre pandemic however now support HPV vaccination post pandemic	1.19 (0.28–4.36)			
SIMD (Scottish Index of Multiple Deprivation) **(Ref = ‘Quintile 1 (Most deprived)’)**	Quintile 2	1.01 (0.59–1.75)	.062	1.10 (0.62–1.94)	.011
	Quintile 3	0.78 (0.45–1.35)		0.61 (0.34–1.08)	
	Quintile 4	1.04 (0.62–1.77)		1.00 (0.57–1.73)	
	Quintile 5	1.21 (0.74–1.98)		1.11 (0.66–1.87)	
Health-related degree/employment **(Ref = ‘No’)**	Yes	2.22 (1.43–3.49)	<.001	2.26 (1.41–3.62)	<.001

Ref, Reference group; OR = odds ratio; aOR, adjusted odds ratio; CI, confidence interval.

### Awareness and knowledge of HPV vaccine and the HPV vaccination programme

Table 2 shows awareness of the HPV vaccine and vaccination programme. HPV vaccine awareness was higher in less deprived areas (86.1% quintile 5 vs. 73.1% quintile 1, *P* < .001) and among females (87.7% vs. 61.0% males, *P* < .001). Vaccination uptake reflected historical availability, with 48.1% of females vaccinated compared to 21.5% of males (*P* < .001). SIMD had no significant effect on uptake (*P* = .517). Awareness of the girl’s vaccination programme was high, especially among females (87.1% vs. 55.7% males, *P* < .001) and in less deprived areas (82.8% quintile 5 vs. 75.5% quintile 1, *P* = .047). Awareness of the boy’s programme was lower overall with females more aware (40.6% vs. 25.0% males, *P* < .001) and a non-significant SIMD trend (42.5% quintile 5 vs. 31.1% quintile 1, *P* = .071). Self-rated HPV vaccine knowledge also varied by SIMD (*P* = .028) and gender (*P* < .001), with females and those from less deprived areas reporting higher knowledge. Despite disparities in awareness, willingness to vaccinate children was high and consistent across all groups (females: 97.1%, males: 94.6%), with no significant SIMD (*P* = .160) or gender differences (*P* = .144). Main motivations were cancer prevention (37.1%), sexual health (21.0%), and vaccine safety (20.2%). Among the few unwilling, reasons included insufficient information (33.3%) and safety concerns (27.0%).

Table 4 shows regression analyses of factors associated with awareness of the boys’ HPV vaccination programme. Awareness was higher among those under 17 (OR = 2.66, 95% CI: 0.95–7.49, *P* = .003) and lower in 25–34 age group (OR = 0.49, 95% CI: 0.31–0.77, *P* = .003). Females were more aware (OR = 2.05, 95% CI: 1.46–2.92, *P* < .001). Having heard of HPV (OR = 4.62, 95% CI: 2.86–7.86, *P* < .001) and HPV vaccine (OR = 11.23, 95% CI: 6.28–22.28, *P* < .001) were the strongest predictors. Health-related background (OR = 2.77, 95% CI: 1.75–4.45, *P* < .001) and supportive post-COVID-19 attitudes (OR = 2.80, 95% CI: 1.44–5.96, *P* = .034) were also significant. In the multivariable model, younger age (aOR = 4.19, 95% CI: 1.3–13.44, *P* < .001), having heard of HPV vaccine (aOR = 10.67, 95% CI: 5.66–20.12, *P* < .001) and health-related background (aOR = 2.60, 95% CI: 1.60–4.23, *P* < .001) remained strong predictors.

**Table 4:** Logistic regression analysis of HPV vaccination programme for boys awareness.

		**Univariable Analysis**		**Multivariable Analysis**	
**Independent Variables**		**HPV vaccination programme for boys awareness** **(‘Yes’ vs ‘No’)**	***P*-value (α = 0.05)**	**HPV vaccination programme for boys awareness** **(‘Yes’ vs ‘No’)**	***P*-value (α = 0.05)**
		**OR (95% CI)**		**aOR (95% CI)**	
Age **(Ref = ‘17–24’)**	Under 17	2.66 (0.95–7.49)	.003	4.19 (1.3–13.44)	<.001
	25–34	0.49 (0.31–0.77)		0.56 (0.33–0.93)	
	35–45	1.15 (0.81–1.65)		1.44 (0.94–2.22)	
	Above 45	0.96 (0.69–1.33)		1.28 (0.84–1.95)	
Gender **(Ref = ‘Male’)**	Female	2.05 (1.46–2.92)	<.001		
Sexual orientation **(Ref = ‘Heterosexual’)**	Bisexual	1.39 (0.85–2.28)	.480		
	Homosexual	0.97 (0.50–1.84)			
	Other	1.36 (0.48–3.70)			
	Prefer not to say	1.97 (0.75–5.31)			
Ethnicity **(Ref = ‘Scottish, English, Welsh, Northern Irish or British’)**	Other White background	1.54 (0.66–3.56)	.651		
	Mixed or Multiple ethnic background	0.56 (0.03–4.40)			
	Asian or Asian British	0.66 (0.29–1.39)			
	Black, African, Caribbean, or Black British	1.68 (0.47–6.10)			
	Others ethnic group	0.84 (0.18–3.21)			
	Prefer not to say	0 (0–Inf)			
Currently in education **(Ref = ‘No’)**	Yes	1.23 (0.94–1.61)	.115		
Employment status **(Ref = ‘Unemployed’)**	Full time	0.66 (0.44–0.99)	.060		
	Part time	0.80 (0.53–1.22)			
	Retired	0.39 (0.19–0.78)			
Heard of HPV **(Ref = ‘No’)**	Yes	4.62 (2.86–7.86)	<.001		
Heard of HPV vaccine **(Ref = ‘No’)**	Yes	11.23 (6.28–22.28)	<.001	10.67 (5.66–20.12)	<.001
Have children **(Ref = ‘No’)**	Yes	1.23 (0.94–1.61)	.146		
Received HPV vaccine **(Ref = ‘No’)**	Yes	1.22 (0.93–1.60)	.239		
Post-COVID-19 pandemic attitudes towards HPV vaccination **(Ref = ‘No—I have remained against HPV vaccination pre and post the pandemic’)**	No—I remain to support HPV vaccination pre and post pandemic	2.80 (1.44–5.96)	.034		
	Yes—I did support HPV vaccination pre pandemic and now do not support HPV vaccination post pandemic	2.51 (0.82–7.68)			
	Yes—I was against HPV vaccination pre pandemic however now support HPV vaccination post pandemic	2.51 (0.57–10.14)			
SIMD (Scottish Index of Multiple Deprivation) **(Ref = ‘Quintile 1 (Most deprived)’)**	Quintile 2	1.07 (0.61–1.91)	.199		
	Quintile 3	1.60 (0.92–2.82)			
	Quintile 4	1.15 (0.66–2.03)			
	Quintile 5	1.64 (0.98–2.77)			
Health-related degree/employment **(Ref = ‘No’)**	Yes	2.77 (1.75–4.45)	<.001	2.60 (1.60–4.23)	<.001

Ref, Reference group; OR, odds ratio; aOR, adjusted odds ratio; CI, confidence interval.

## Discussion

### Main findings of this study

This study reveals a significant gap in public awareness of HPV-related cancers beyond cervical cancer and the Scottish HPV vaccination programme for boys. While awareness of the HPV-cervical cancer link was high across socioeconomic groups, a gender gap remained, with more females than males identifying this association. The proportion who recognized HPV as the main risk factor for cervical cancer was low, particularly among males, indicating that recognition does not equate to understanding HPV’s causal role. Knowledge of other HPV-related cancers was lower, with anal cancer (<20%) and oropharyngeal cancer (<10%) showing the lowest recognition despite oropharyngeal cancer being the predominant male HPV-related cancer.^16^ These cancer-type-specific gaps persisted regardless of gender or SIMD. When awareness was measured as a combined indicator (≥1 male HPV-related cancer), gender and SIMD differences emerged, showing aggregated measures yield different disparity patterns than site-specific analyses. This highlights the importance of analysing awareness by individual cancer type to avoid masking inequalities and to inform targeted public health messaging, as some awareness deficits may be universal and unlikely to be addressed by interventions targeting specific demographics. Across most other survey items, males were consistently less aware than females. Notably, only 48.9% knew boys are included in Scotland’s school-based vaccination programme implemented in 2019, a figure comparable to pre-rollout estimates from England.^17^

These findings likely reflect the longstanding framing of HPV as a women’s health issue, shaped by early public health messaging focused almost exclusively on cervical cancer prevention and vaccination for girls.^18^ Non-cervical HPV-related cancers, particularly those affecting men, received little attention in public campaigns or school-based education.^3^ This may explain persistent gender gaps in HPV awareness, even after rollout of gender-neutral vaccination. Men’s lower likelihood of seeking health information or engaging in preventive behaviours may further contribute to disparities in HPV awareness.^19^ In this study, under-17 s demonstrated greater awareness of the boys’ vaccination programme, suggesting that updated school-based consent material introduced before rollout improved awareness among newer cohorts.

A further significant finding was the socioeconomic gradient in HPV awareness. This aligns with Scottish data showing socioeconomic disparities in HPV vaccine uptake^6^ and with broader evidence linking deprivation to lower cancer knowledge and reduced engagement with preventive health behaviours.^20^^,^^21^ However, this study extends the evidence by demonstrating inequalities in male-specific and non-cervical cancer awareness. The SIMD effect was less consistent when analysed by individual cancer site, reinforcing that gaps in oropharyngeal and anal cancer awareness are pervasive across socioeconomic strata. These disparities emerged despite willingness to vaccinate as over 92% of participants indicated they would vaccinate their children, even when knowledge of non-cervical cancers was limited. This ‘knowledge-action gap’ challenges health behaviour models that assume awareness precedes action.^22^ Instead, it suggests that trust in health systems, perceived safety of school-based vaccination and habitual acceptance of childhood immunization may be more influential than disease knowledge.^23^ The link between post-COVID-19 vaccine confidence and HPV vaccine awareness further supports the importance of maintaining public trust to sustain universal uptake.

### What is already known on this topic

Previous research has shown that public awareness of HPV focuses on its relationship with cervical cancer, with limited recognition of other cancers and uncertainty about boys’ vaccine eligibility.^24^^,^^25^ While the expansion of HPV vaccination programmes to include boys has occurred in several countries, educational efforts have not kept pace with policy changes. Studies also show that females are more likely than males to be aware of the HPV-cervical cancer link; however, findings are mixed for non-cervical, male-associated cancers.^11^^,^^25^ Socioeconomic disparities in HPV awareness and uptake have also been documented across both universal and insurance-based healthcare systems, including in France^20^ and the USA.^21^ However, most research has focused on cervical cancer or general awareness, with few examining whether knowledge of non-cervical and male-associated cancers varies by socioeconomic status: a gap our study addresses.

Thus, despite rising incidence rates of oropharyngeal, anal and penile cancers, public understanding of HPV’s broader disease burden remains limited, particularly among men.^3^ In Scotland, recent declines in HPV vaccine uptake, especially among boys, raise concerns that public awareness and trust is weakening.^6^ Although participants in this study generally expressed strong support for HPV vaccination, citing cancer prevention and vaccine safety as key motivations, these messages may not be reaching all groups. In particular, health communication may be failing to engage socioeconomically disadvantaged populations, compounding inequalities in HPV-related cancer prevention.

### What this study adds

This study provides insights into persistent knowledge gaps regarding HPV-related cancers affecting males, despite Scotland’s implementation of a gender-neutral, school-based vaccination programme. It is among the first to document socioeconomic inequalities in awareness of male-specific and non-cervical HPV-related cancers using SIMD and contributes to the international evidence base by addressing these gaps in a universal healthcare setting with high vaccination coverage, while also identifying cancer-type-specific gaps that persist across demographic groups. The uniformly low recognition of oropharyngeal and anal cancer suggests these sites are underrepresented in public education campaigns. Aggregated male-specific awareness measures showed gender and SIMD effects, but these were absent for individual cancers, highlighting the importance of site-specific analysis to address knowledge deficits.

The study identifies a clear ‘knowledge-action gap’ where high vaccination acceptance exists despite limited awareness of HPV-related risks. This challenges conventional health behaviour models suggesting uptake can be sustained through strong delivery infrastructure and public trust, even when specific knowledge is low. While Scotland performs favourably compared to England (72.9% uptake girls, 67.7% boys)^17^ and Europe (59.2% average uptake),^3^ these findings highlight a missed opportunity to improve awareness and reduce inequalities. Embedding equity and trust into HPV communication strategies, for example through co-designed materials for deprived communities, integration into school curricula and use of trusted local outreach channels, may help ensure that both knowledge and uptake are strengthened.

### Limitations of this study

While this study provides valuable insights into HPV awareness and knowledge of male and female-associated cancers across socioeconomic groups in Scotland, several limitations are acknowledged. Its cross-sectional design limits causal inference and self-reported data may be subject to recall or social desirability bias. The sample overrepresented young females and individuals with higher education in health-related fields. Nearly half of respondents were aged 17–24. This overrepresentation suggests our sample had higher baseline awareness of HPV and vaccination than the wider Scottish population, potentially biasing estimates upward. Although this introduces sampling bias and limits generalizability, this age group is the most sexually active and at greater risk for HPV infection,^26^ making it a relevant public health focus. Despite these limitations, the study contributes to the limited evidence base on socio-demographic influences on awareness of non-cervical HPV-related cancers and vaccination.

## Conclusions

Despite Scotland’s robust school-based vaccine delivery and high willingness to vaccinate, awareness of HPV-related cancers affecting males and the boys’ vaccination programme remains limited, indicating deficiencies in public understanding of HPV’s disease burden and contributing new evidence to international understanding of how gender and socioeconomic disparities persist despite successful vaccination programmes. Public health strategies should prioritize integrating gender-neutral HPV education and developing targeted, culturally sensitive outreach initiatives, essential to improving literacy, reducing inequalities and reinforcing confidence.

## Notes

**Huyen Nguyen-Thanh**, Pharmacist

**Leah Marshall**, Biochemistry and Pharmacology Graduate

**Hannah Wilson**, Biochemistry and Pharmacology Graduate

**Kirsty Stewart**, Health Improvement Manager, Public Health Scotland

**Kimberley Kavanagh**, Professor of Statistics and Epidemiology

**Annette Sorensen**, Teaching Fellow
